# Acquired generalized idiopathic anhidrosis ‒ rare case in a Brazilian patient^⋆^

**DOI:** 10.1016/j.abd.2020.10.025

**Published:** 2022-06-01

**Authors:** Maísa Aparecida Matico Utsumi Okada, Letícia Santos Dexheimer, Renan Rangel Bonamigo, Renata Heck

**Affiliations:** a Ambulatory of Sanitary Dermatology, Secretaria Estadual de Saúde do Rio Grande do Sul, Porto Alegre, RS, Brazil; b Faculdade de Medicina, Universidade Federal do Rio Grande do Sul, Porto Alegre, RS, Brazil

Dear Editor,

Acquired generalized idiopathic anhidrosis (AGIA) is a rare disease without a defined etiology, non-related to dysautonomia or neurological abnormalities.^1^ It is an uncommon condition, with approximately 100 cases described, the majority in Asia, affecting men aged 20 to 30 years 80% of the time.^1^ Considering the predominance of cases described in Asia, this case report describes an unprecedented case in Brazil.

A 20-year-old military male, of non Asian descent, without comorbidities, had developed diffuse anhidrosis for eight months, not affecting the palmoplantar and axillary regions, associated with multiple small normochromic papules, with an erythematous base, affecting the face, trunk, back region and upper limbs (Fig. 1). The rash onset occurs after physical activity, exposure to heat, or emotional stress and has a short duration, showing spontaneous resolution and is associated with a burning sensation. The symptoms are worse in summer, and there is no family history of sweating disorder or urticaria. The diagnosis of anhidrosis was confirmed by Minor's test (Fig. 2), and the histopathological examination revealed a decrease in the number of eccrine glands and the presence of a periglandular lymphocytic infiltrate. The laboratory tests showed a slightly elevated carcinoembryonic antigen (CEA) level (7.5 ng/mL), normal IgE levels, negative ANA, Anti-SSA, and Anti-SSB, with normal results for the blood count, renal and thyroid function, and fasting glucose tests. The magnetic resonance imaging of the skull showed no abnormalities. There was a partial and gradual improvement of the rash with prednisone 1 mg/kg/day, orally.

**Figure 1:**
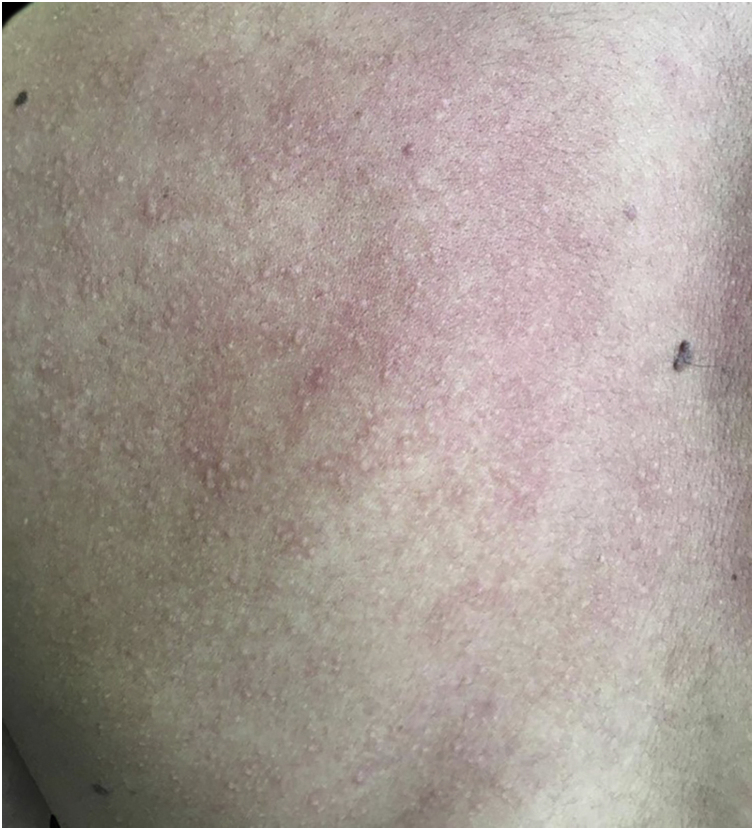
Several normochromic papules and erythema induced by physical activity.

**Figure 2:**
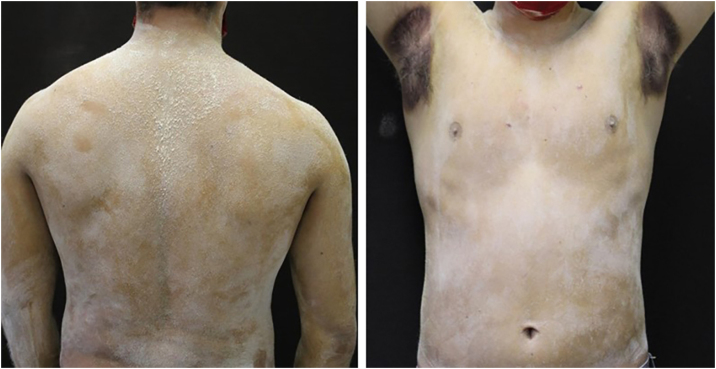
Minor's test demonstrating preserved sweating only in the axillary region after strenuous physical activity.

AGIA clinically presents as the absence of sweating after stimulation. The symptoms of heat intolerance and cholinergic urticaria are associated, corroborating the predominance in individuals whose work activity involves exposure to heat. Preserved palmoplantar and axillary sweating is justified by the adrenergic innervation and the predominance of apocrine sweat glands, respectively.^2^

The diagnosis is clinical, and pathological examination may reveal the presence of perieccrine lymphocytic inflammatory infiltrates.^1^ CEA is expressed in normal eccrine glands, and the increase in CEA levels correlates with the body surface area affected by anhidrosis and can be used to monitor disease activity.^3^

The differential diagnosis of anhidrosis includes congenital and acquired causes (Table 1). Three subtypes of AGIA have been described: eccrine gland dysfunction, sudomotor axis neuropathy, and idiopathic pure sudomotor axis dysfunction, which is the most common subtype and is found in cases where cholinergic urticaria is associated.^1^, ^4^ Its pathogenesis is not well understood^1^, ^4^ but it is known that there is a reduction in muscarinic receptors in the eccrine glands, as evidenced by immunohistochemistry.^5^ A reduction in the enzyme acetylcholinesterase is also found, resulting in excess acetylcholine in the synaptic cleft. The latter binds to muscarinic receptors in mast cells, inducing degranulation and wheal formation. It is postulated that the expression of cytokines CCL2/MCP-1, CCL5/RANTES, and CCL17/TARC is increased in glandular cells, recruiting lymphocytes that would affect the expression of muscarinic receptors and acetylcholinesterase.^5^

**Table 1:** Causes of anhidrosis.

Congenital	Acquired
Anhidrotic ectodermal dysplasia	***Idiopathic***
Insensitivity to pain and anhidrosis	Acquired generalized idiopathic anhidrosis
Rapp Hodgkin Syndrome Fabry disease	***Secondary***
	*Central neurological diseases*
	Parkinson's disease
	Ischemia
	Lewy bodies dementia
	Multiple sclerosis
	Tumors
	Peripheral neurological diseases
	Diabetes mellitus
	Leprosy
	Alcoholism
	Collagenoses
	Sjogren's syndrome
	Medications
	Psychotropic agents
	Anticholinergics

Despite reports of therapeutic success with corticosteroid use, there are no clinical studies to prove its efficacy. There is no consensus on the method of administration, dose, and duration of treatment.^1^ If corticosteroid therapy fails, cyclosporine, intravenous immunoglobulin, and omalizumab can be used.^1^, ^4^

## Financial support

None declared.

## Authors' contributions

Maisa Aparecida Matico Utsumi Okada: Design and planning of the study; data collection, or analysis and interpretation of data; drafting of the manuscript.

Letícia Santos Dexheimer: Critical review of important intellectual content; approval of the final version of the manuscript.

Renan Rangel Bonamigo: Critical review of important intellectual content; approval of the final version of the manuscript.

Renata Heck: Critical review of important intellectual content; approval of the final version of the manuscript.

## Conflicts of interest

None declared.
